# Supplementary material for: Active maintenance of proton motive force mediates starvation-induced bacterial antibiotic tolerance in Escherichia coli
Source: Commun Biol. 2021 Sep 14;4:1068. doi: 10.1038/s42003-021-02612-1 (PMC8440630; doi:10.1038/s42003-021-02612-1)
Supplement: Supplementary file 2 — Supplementary Information [file 42003_2021_2612_MOESM2_ESM.pdf]

Supplementary Information

Active Maintenance of Proton Motive Force Mediates Starvation-induced Bacterial Antibiotic Tolerance in *Escherichia coli*

Miaomiao Wang[1,2†], Edward Wai Chi Chan[1†], Yingkun Wan[2], Marcus Ho-yin Wong[1], Sheng Chen[2*]

[1] State Key Laboratory of Chemical Biology and Drug Discovery, Department of Applied Biology and Chemical Technology, The Hong Kong Polytechnic University, Hung Hom, Kowloon, Hong Kong.

[2]Department of Infectious Diseases and Public Health, Jockey Club College of Veterinary Medicine and Life Sciences, City University of Hong Kong, Kowloon, Hong Kong.

*Corresponding authors: Sheng Chen, Tel: (852)-3442-5782; Email: shechen@cityuu.edu.hk;

†Contributed equally.

Keywords: Antibiotic tolerance, Starvation, PMF, Protonophore

Supplementary Figures

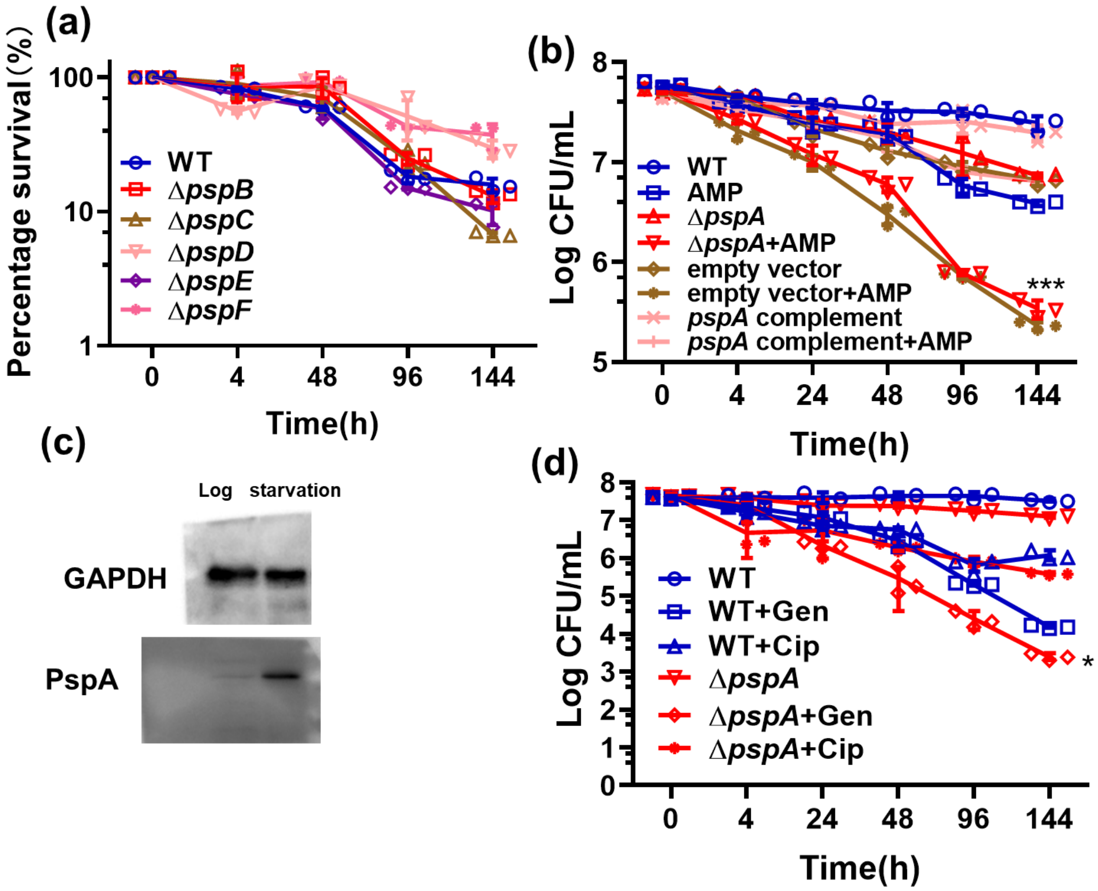

Supplementary Figure 1. Bacterial antibiotic tolerance was negatively affected by *pspA* deletion. (a) Relative tolerance ratio of the wild type strain and *psp* mutants calculated by comparing the size of bacterial population that survived treatment with ampicillin at 100μg/mL for 144hrs upon starvation for 24hrs to those without ampicillin treatment. (b) Complementation of Δ*pspA* with plasmid-borne copies of *pspA* restored tolerance to ampicillin. Wild type and Δ*pspA* are included as control. P value was tested between Δ*pspA* and Δ*pspA* +AMP at 144hr. (c) The original blot image which related to Fig. 1c. (d) The size of population of wild type and Δ*pspA* upon starvation for 24hrs followed by treatment with 10μg/mL gentamicin (Gen) or 0.5μg/mL ciprofloxacin (Cip) for 144hrs. n=3 biologically independent experiments. P value was tested between WT+Gen and Δ*pspA*+Gen at 144hr.* indicate a P value of <0.05, ***indicate a P value of <0.001by two-tailed Student's test. Error bar represents standard deviation.

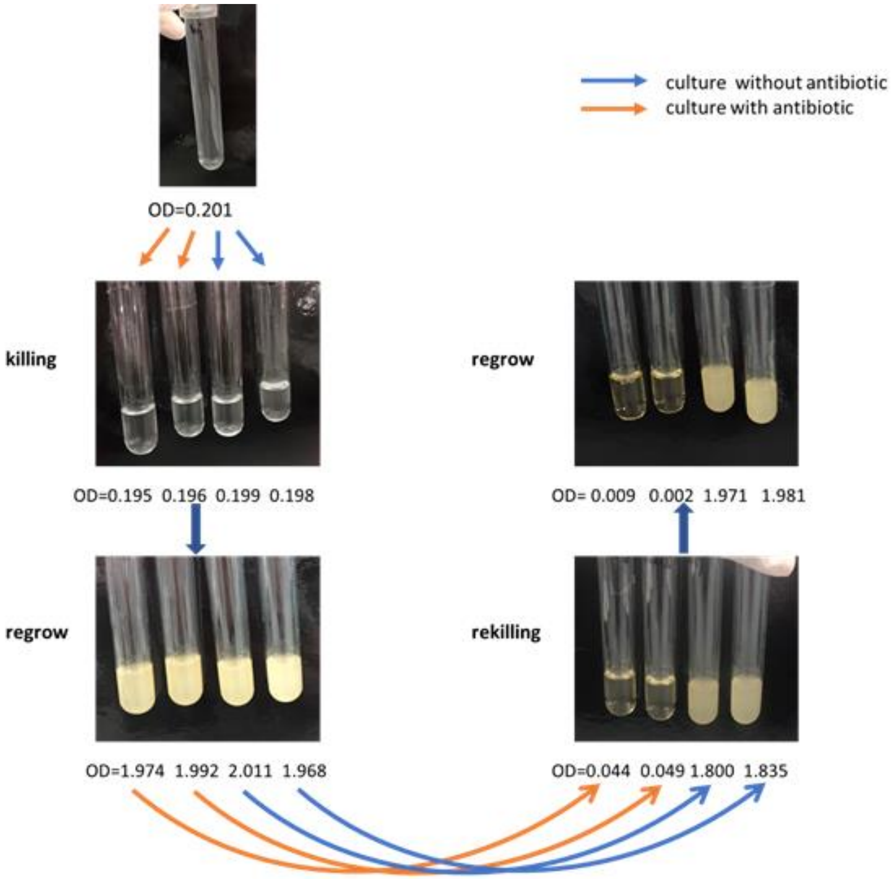

Supplementary Figure 2. Assessment of antibiotic susceptibility of bacterial sub-population exhibiting starvation-induced antibiotic tolerance. In order to confirm that the antibiotic tolerance phenotype observed in the tolerance assay was not due to existence of a resistant sub-population, bacteria subjected to nutrient starvation for 24hrs were split into two portions, one was treated with 100μg/mL ampicillin for 4hrs to obtain antibiotic tolerant sub-population, and the one without antibiotic was set as control. The tolerant sub-population was then collected by centrifugation, followed by re-suspension and dilution in fresh LB and incubation at 37°C to induce regrowth. Fresh bacterial culture derived from this tolerant sub-population was subjected to antibiotic susceptibility tests, with results confirming that offsprings of such sub-population remained susceptible to the test agent. Two biological replicates were tested.

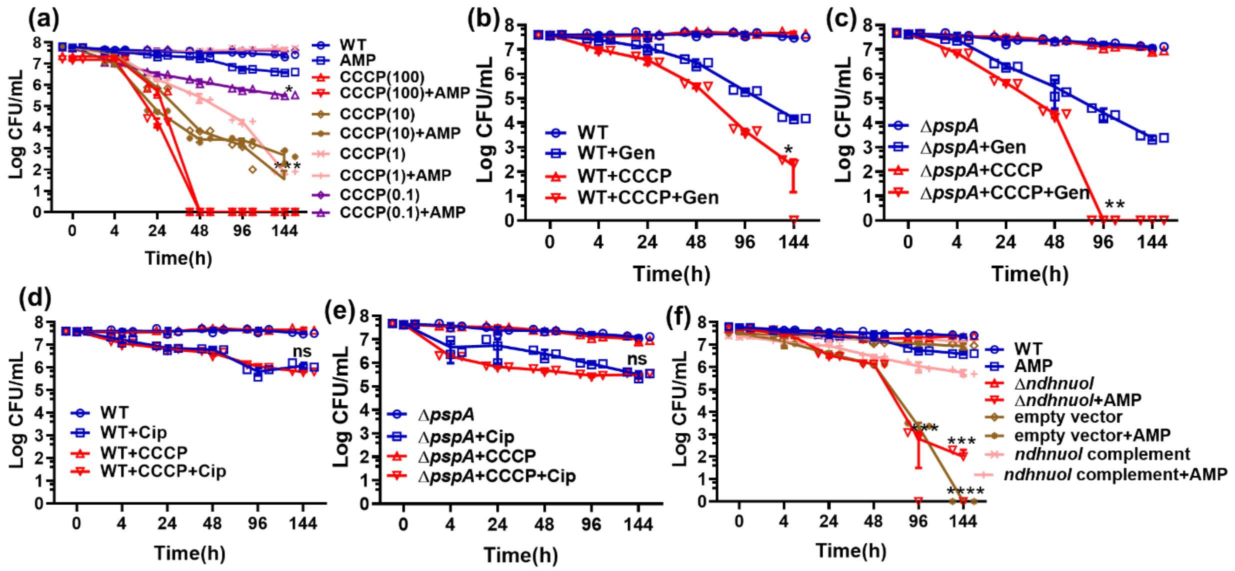

Supplementary Figure 3. PMF dissipation negatively affects bacterial tolerance to ampicillin. (a) The size of the bacterial population of the wild type *E. coli* strain BW25113 that survived at different time points upon starvation for 24hrs, followed by treatment with ampicillin, CCCP or a combination of these two compounds. CCCP (100), 100μM CCCP; CCCP (10), 10μM CCCP; CCCP (1), 1μM CCCP; CCCP (0.1), 0.1μM CCCP. P values were tested between CCCP and CCCP+AMP with the same concentration. (b-e) The size of the bacterial population of the wild type or Δ*pspA* strains that survived at different time points upon starvation for 24hrs, followed by treatment with gentamicin (10μg/mL), ciprofloxacin (0.5μg/mL), CCCP (1μM) or a combination of such compounds. P values were tested between Gen/Cip and CCCP+ Gen/Cip at indicated time points. (f) Complementation of Δ*ndh*Δ*nuoI* with plasmid-borne copies of *ndh and nuoI* restored tolerance to ampicillin. Wild type and Δ*ndh*Δ*nuoI* are included as control. P values were tested between Δ*ndh*Δ*nuoI* and Δ*ndhnuoI* +AMP, empty vector and empty vector +AMP at indicated time points. ns indicates no significance, * indicates a P value of <0.05, ** indicates a P value of <0.01, ***indicates a P value of <0.001, ****indicates a P value of <0.0001by two-tailed Student's test. Error bar represents standard deviation.

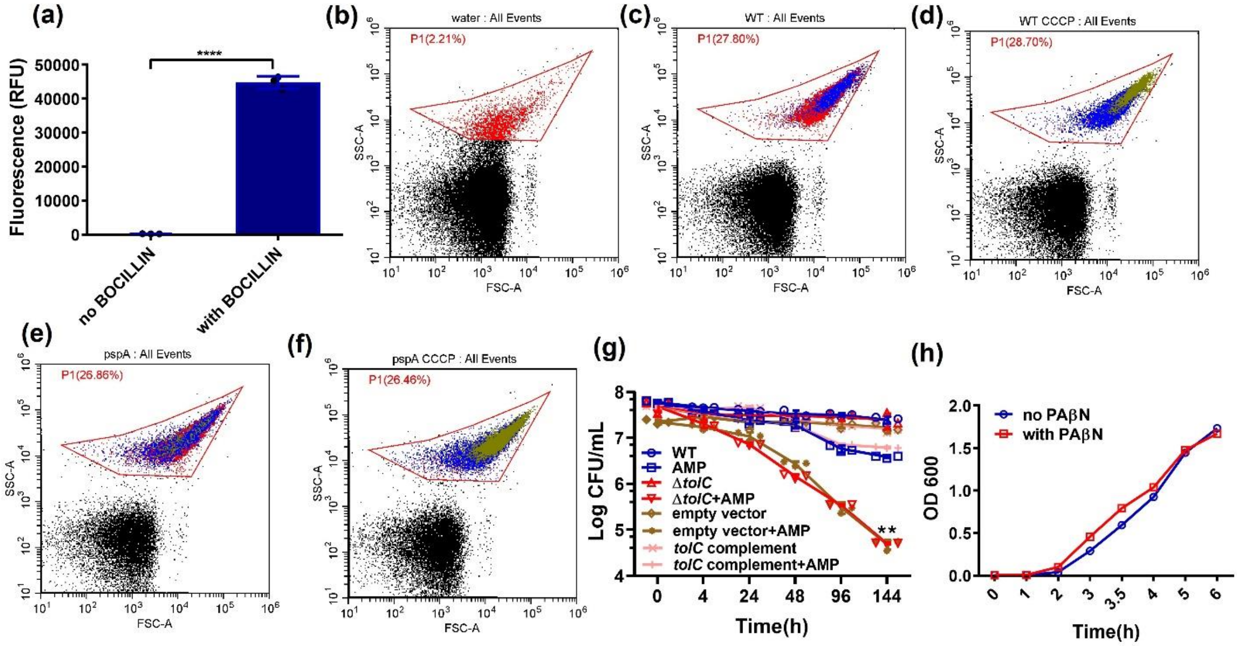

Supplementary Figure 4. Evaluation of intracellular fluorescent β-lactam amount and the effect of efflux pumps upon bacterial tolerance. (a) CCCP does not affect the level of fluorescence exhibited by BOCILLIN. The fluorescence signal of bacterial population treated with CCCP only (no BOCILLIN) was measured and compared with those treated with both CCCP and BOCILLIN (with BOCILLIN). (b-f) FSC-SSC profiles of BOCILLIN stained wild type and Δ*pspA* cells with or without CCCP(1μM). P1 gate was determined as the bacteria sector since the percentage of P1 in samples (~30%) is much higher than that in water (~2%). (g) Complementation of Δ*tolC* with plasmid-borne copies of *tolC* restored tolerance to ampicillin. Wild type and Δ*tolC* were included as control. P value was tested between Δ*tolC* and Δ *tolC*+AMP at 144hr. (h) Growth rate of wild type strain in the presence and absence of PAβN (100μM). The results show that PAβN does not inhibit bacterial growth. ** indicates a P value of <0.01, **** indicates a P value of <0.0001by two-tailed Student's test. Error bar represents standard deviation.

Supplementary Table 1. MIC of gene knockout strains

**Strain**	**Ampicillin MIC(μg/mL)**	**Strain**	**Ampicillin MIC(μg/mL)**
BW25113	8	Δ*pspD*	8
Δ*pspA*	8	Δ*pspE*	8
Δ*pspB*	8	Δ*pspF*	8
Δ*pspC*	8	Δ*tolC*	8

Supplementary Table 2. MIC of ampicillin for bacterial strains of various species.

Bacterial strains	Ampicillin
	MIC (μg/ml)
E. coli BW25113	8
S. aureus ATCC29213	2
K. pneumoniae ATCC13883	512
A. baumannii ATCC19606	128
P. aeruginosa PAO1	1024
S. typhimurium PY01	1

Supplementary Table 3. *E. coli* strains used in this study

Strain	Genotype	Reference
BW25113	*lacI*^{q} *rrnB*$_{T14}$Δ*lacZ*$_{WJ16}$ *hsdR514*Δ*araBAD*$_{AH533}$Δ*rhaBAD*$_{LD78}$	[1]
JW1297	BW25113 *pspA*::Km^{r}	This study
JW1298	BW25113 *pspB*::Km^{r}	This study
JW1299	BW25113 *pspC*::Km^{r}	This study
JW1300	BW25113 *pspD*::Km^{r}	This study
JW1301	BW25113 *pspE*::Km^{r}	This study
JW1296	BW25113 *pspF*::Km^{r}	This study
JW1095	BW25113 *ndh*::Km^{r}	This study
JW2276	BW25113 *nuoI*::Km^{r}	This study
JW5503	BW25113 *tolC*::Km^{r}	This study

Supplementary References:

1. Baba, T. *et al.* Construction of Escherichia coli K-12 in-frame, single-gene knockout mutants: the Keio collection. *Mol Syst Biol* **2**, 2006 0008 (2006).
