# Supplementary material for: Active maintenance of proton motive force mediates starvation-induced bacterial antibiotic tolerance in Escherichia coli
Source: Commun Biol. 2021 Sep 14;4:1068. doi: 10.1038/s42003-021-02612-1 (PMC8440630; doi:10.1038/s42003-021-02612-1)
Supplement: Supplementary file 3 — Description of Supplementary Files [file 42003_2021_2612_MOESM3_ESM.pdf]

Description of Additional Supplementary Files

File name: Supplementary Data 1

Description: 62 shortlisted genes whose expression level was found to be up-regulated by three folds or more in RNA-Seq upon starvation for 24hrs.

File name: Supplementary Data 2

Description: Source data presented in the main figures". Each sheet listed one main figure's source data, for example, sheet named "Fig 1" listed all the source data presented in Fig 1.
